# Cost-utility of a walking programme for moderately depressed, obese, or overweight elderly women in primary care: a randomised controlled trial

**DOI:** 10.1186/1471-2458-8-231

**Published:** 2008-07-08

**Authors:** Narcis Gusi, Maria C Reyes, Jose L Gonzalez-Guerrero, Emilio Herrera, Jose M Garcia

**Affiliations:** 1 Faculty of Sports Sciences, University of Extremadura, Cáceres, Spain; 2 Geriatric Unite, Hospital of Cáceres, Cáceres, Spain; 3 Health System of Extremadura, Junta de Extremadura, Mérida, Spain

## Abstract

**Background:**

There is a considerable public health burden due to physical inactivity, because it is a major independent risk factor for several diseases (e.g., type 2 diabetes, cardiovascular disease, moderate mood disorders neurotic diseases such as depression, etc.). This study assesses the cost utility of the adding a supervised walking programme to the standard "best primary care" for overweight, moderately obese, or moderately depressed elderly women.

**Methods:**

One-hundred six participants were randomly assigned to an interventional group (n = 55) or a control group (n = 51). The intervention consisted of an invitation, from a general practitioner, to participate in a 6-month walking-based, supervised exercise program with three 50-minute sessions per week. The main outcome measures were the healthcare costs from the Health System perspective and quality adjusted life years (QALYs) using EuroQol (EQ-5D.)

**Results:**

Of the patients invited to participate in the program, 79% were successfully recruited, and 86% of the participants in the exercise group completed the programme. Over 6 months, the mean treatment cost per patient in the exercise group was €41 more than "best care". The mean incremental QALY of intervention was 0.132 (95% CI: 0.104–0.286). Each extra QALY gained by the exercise programme relative to best care cost €311 (95% CI, €143–€394). The cost effectiveness acceptability curves showed a 90% probability that the addition of the walking programme is the best strategy if the ceiling of inversion is €350/QALY.

**Conclusion:**

The invitation strategy and exercise programme resulted in a high rate of participation and is a feasible and cost-effective addition to best care. The programme is a cost-effective resource for helping patients to increase their physical activity, according to the recommendations of general practitioners. Moreover, the present study could help decision makers enhance the preventive role of primary care and optimize health care resources.

**Trial Registration:**

[ISRCTN98931797]

## Background

Physical inactivity is a considerable public health burden, because it is a major independent risk factor for several diseases (e.g., type 2 diabetes, cardiovascular disease, moderate mood disorders neurotic diseases such as depression, etc.) [1,2]. It is also associated with a high prevalence of musculoskeletal disorders [3]. Moderate depression and being overweight are among the most common and expensive public health problems and leading reasons for consultation in primary care [4,5]. A large proportion of health care resources – including office visits, hospitalisation, and medication – are required by the elderly.

General practitioners usually recommend increasing physical activity because even such moderate increases have been shown to improve the quality of life in older adults [6,7] and elderly are interested in sports, walking and health [8]. However, rates of physical inactivity are substantial in elderly women [9-12]. Therefore, physical activity should be promoted, especially in elderly populations. The National Institute for Health and Clinical Excellence (NICE) of the United Kingdom established the need to study the clinical- and cost-effectiveness of pedometers and exercise referrals because there is limited evidence to recommend these interventions based on studies of walking and cycling, especially in trials longer than 12 weeks [13]. Bjorgaas et al. recently reported that the use of a pedometer with weekly monitoring by nurses is effective in physically active patients with diabetes mellitus, but this activity was not sustained in relatively inactive patients with diabetes mellitus type 2 [14].

Traditional community-based exercise referral schemes involve referral from a primary care practitioner to a 10–12 week exercise programme run by local leisure services. These programmes succeed in the short-term [15,16] but are limited in terms of sustainability and impact on health outcomes [15]. From a health economics perspective, just the assessment and advice from an exercise specialist may be appropriate to initiate action. However, the maintenance of increased activity requires further support (technical or societal support by phone or peers). This need was demonstrated in a study that showed that the proportion of participants who maintained an active lifestyle three months after the cessation of 10-week programmes ranged between 7.5% in the advice-only group and 11–14% in the leisure centre group or walking group [17]. Walking appears to be as effective as leisure centre classes and is less expensive [17] but there is a lack of knowledge about walking programmes, especially those that add strengthening and stretching exercises. Wormald et al. [18] indicated that the success of the service was highly dependent upon the exercise advisor and that traditional schemes should be broadened to encompass everyday lifestyle activities. However, as health system resources are limited, the decision-maker frequently selects the strategies adopted based on the lowest cost per quality-adjusted life-years (QALY). Cost utility is the ratio of incremental effectiveness of one strategy compared to another (e.g., standard medical practice) measured in QALYs divided by the incremental cost.

The cost and effectiveness of the patient recruitment strategy are among the most relevant determinants of the cost utility of physical activity promotion. However, further research on recruitment from practice-based populations is required [19,20]. In addition, among the few cost-utility analyses of exercise interventions in primary care, most involved patients younger than 60 years of age who suffered heart disease or back pain. Thus, the effectiveness of exercise programmes with overweight or moderately depressed, elderly females remains largely unknown.

The purpose of this study was to assess the cost utility of adding to the standard "best care" a supervised walking programme that also included strengthening and stretching exercises The patients involved were elderly females who were overweight or moderately depressed.

## Methods

### Recruitment

Four general practices in Cáceres (Spain) were recruited to participate in the study. Practices were selected at random, from those with two to five partners, and these practices were not previously conducting an exercise programme or exercise prescription scheme. Of five general practices approached, four agreed to participate. The distances between practices allowed the grouping of participants into two exercise groups.

The population of the catchment area comprised elderly women who consulted one of the family physicians who practiced in one of four public primary care centres participating in this study. Eligible women were aged 60 years and older, suffered from either moderate depression or were overweight, and were capable of walking for more than 25 minutes. Women who scored 6 to 9 points in the 15-item Geriatric Depression Scale were considered moderately depressed [21]. Women who had a body mass index (BMI) of 25 to 39.9 kg/m^2 ^were considered overweight (overweight or obese type I or II). Patients were excluded if they had poor health (severe obesity or major depression), a debilitating medical condition or a known unstable cardiac condition, attention or comprehension problems (e.g., Alzheimer's, apraxia, global aphasia, and other types of dementia or psychopathology), or the intention of leaving the region. No patients were excluded following enrolment.

Physicians advised eligible women on the walking programme, called "Exercise Looks After You", as part of a complete health, fitness and nutritional assessment. In fact, the participating physicians previously included general advice in their routine so the additional time and effort required was kept to a minimum because of the practitioners' busy schedules. All participants provided informed consent in writing, and the study was approved by the Bioethics Committee of the University according to the Declaration of Helsinki.

### Study design

**Figure 1:**
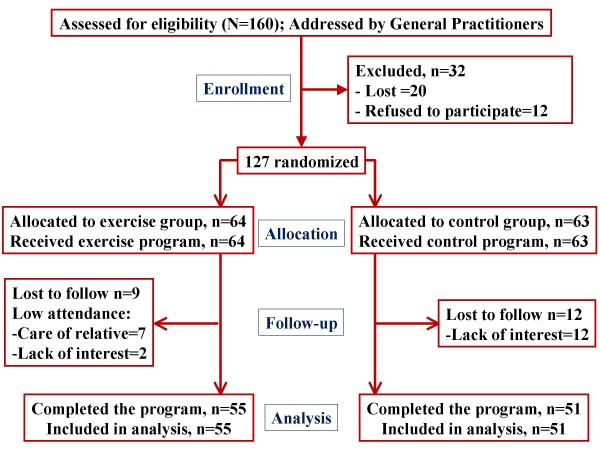
Flow-chart of participants throughout trial.

Medical practitioners spent 2 weeks at each practice referring patients to researchers. A research assistant, who did not participate in the current investigation, randomised participants to either an intervention group or control group, according to a random numbers table. The flow-chart of participants is presented in Figure 1. Consistently, medical practitioners did not know which group patients were randomised to prior to their exercise referral, and they did not interact with the research team throughout the trial. Researchers evaluated participants at baseline and after the 6-month programme.

### Interventions

Two alternatives: best care in general practice and the addition of a walking programme were compared. The research group educated practice teams in "active management" by identifying eligible patients and giving exercise advice.

#### Exercise programme

This intervention was a walking programme that consisted of supervised walks with a group in a public park or forest tracks. The qualified exercise leaders had expertise in fitness testing and the supervision of physical activity in groups, and had been graduated from university programs in sport sciences. These leaders instructed and trained the intervention group for 50 minutes, three times per week over a 6-month period. Each session consisted of walking alternating with specific exercises, as follows: 5 minutes of joint mobility (8–12 easy rotations at the neck, shoulder, hip and ankle and 8–12 easy flexion-extensions of the knee, wrist and elbow); 15 minutes of brisk-walking; 5 minutes of strengthening (8–12 flexion-extension of arms against a wall, 8–12 spine flexion with elevation of alternating knees, in a standing position) and stretching (hamstrings and shoulders [trying to touch the fingers on the upper-back]); 20 minutes of brisk-walking including 20 foot-steps and 50 hand-claps to provide additional mechanical impact. In addition, simple nutritional advice was provided. The technicians' schedule included 10 hours per week from Monday to Friday. This programme was designed without reference to any explicit behavioural model or theory; rather, it was intended as a pragmatic intervention that could be easily organised for a large population by a public health agency. The leader neither recommended nor advised against practice between sessions. Socialising within the group was encouraged.

#### Control group

Patients in the control group received the best care in general practice, which consisted of routine care and a recommendation of physical activity. These participants in the control group underwent fitness testing by the research team.

### Data collection

Participants completed questionnaires, including the EQ-5D health status instrument [22] at the beginning of the programme and after the 6-month programme. Over the same period, health care was recorded, including hospital stays, drug usage, secondary care, primary care and physical therapist visits; health care from private resources and within the National Health System was recorded.

### Unit costs

Participants were retired and living close to practices, and were recruited in a previously arranged consultation without considering the current project; thus, we decided to perform an economic analysis from the health service perspective. This decision was based on the assumption that marginal societal costs were negligible (there were no additional costs of displacement, and participants did not lose work-hours). In fact, this analysis were recommended by NICE to contribute to health policy for an expensive condition. The unit costs are expressed in Euros at 2005 prices. Participants' follow-up periods were between January and July of the same year. Costs were not adjusted or discounted, as we solely focused on effects over a 6-month period. The cost of the programme was based on the salary of a graduate in sports science in health promotion; this salary was published in the official 2005 bulletin of the regional government. The cost of the programme did not include other possible costs because the clinical analysis did not show statistically significant changes in the use of the National Health System (medication, consultation, etc.) and there was no difference between groups regarding assessment and advice from a physician. In addition, we did not hire any facility (but used public parks or forest tracks), and the recruitment did not require any additional time by the practitioner.

### Health outcomes

The EQ-5D [22] was used to assess five dimensions of health related quality of life (HRQOL): (1) mobility; (2) self-care; (3) daily activities; (4) pain and discomfort; and (5) anxiety or depression. The scale of dimensions is from 1 to 3 (no problems, some problems, or extreme problems). Using a combination of these dimensions, a total of 243 possible health states exist. Each health state has been previously defined using the time trade-off method of utility analysis based on the response of a sample of the Spanish population [23]. This total score of utility was scaled from 1 = a fully functional quality of life to 0 = death. The QALYs that the participants experienced over the 6-month period was estimated by calculating "areas under (health utility) curves" [24]. To avoid bias, data were adjusted for differences in baseline EQ-5D scores by regression analysis [25]. The State-Trait Anxiety Inventory (STAI) [26], was used to evaluate anxiety by using 20 items with a scale of 0 to 3. The Trait Anxiety index is the sum of these 20 scores expressed as a percentage. The fifth questionnaire, the Geriatric Depression Scale [27], includes 15 questions to evaluate the level of depression from 0 (no depression) to 15 (the worst level of depression). A score of 5 or higher indicates the presence of depression.

### Sample size

The primary outcome was the EQ-5D utility. The required sample size was calculated with the Spanish EQ-5D data set for a hypothetical study comparing two groups with a significance level alpha (0.05) and 80% of the power needed for a minimal clinically relevant difference of 0.1 [28]. The required sample ranges between 60 participants extracted from general population and 100 extracted from critically ill participants. We selected at least 120 participants to exceed the higher number by 20%, thus allowing for potential drop-outs.

### Statistical analysis

The statistical analysis was performed by researchers who had not participated in the data collection or implementation of the exercise programme. Normality of data was initially tested using the Kolgomorov-Smirnov test using the correction of Lillifors. The effects of programme on anxiety, depression and Body Mass Index were tested using analyses of variance (ANOVA) for continuous variables. Age-adjusted analyses of covariance were used to compare, between the groups, the changes in measured variables over time (from the beginning of the programme to 6 months). For all tests the significance level was set at p < .05. The analyses were done using SPSS 15.0 (SPSS Inc. Chicago, USA).

### Cost utility analysis

Most of the participants who dropped out declined to be tested after the period of intervention, so the research team performed a non-intent-to-treat analysis. We first estimated the incremental mean costs of the programme and the mean QALYs gained by the two treatment alternatives. Secondly, the incremental cost effectiveness ratio for the intervention was calculated by dividing the incremental costs by incremental QALYs.

To report the uncertainty due to sampling variation, we calculated the 95% confidence interval (CI) using the non-parametric bootstrapping technique (1000 replicates re-sampled with replacement from treatment and control populations) and plotted a cost effectiveness acceptability curve [29,30]. This curve estimates the probability that the intervention is cost effective compared with the alternative, across the range of values that decision makers pay to achieve an additional QALY. The "investment ceiling" is the level of spending that should not be exceeded, even assuming unlimited funding. For the health care system in Spain, the 2005 adjusted investment ceiling was set at €34729/QALY [31]. Decision makers should compare this upper limit of acceptable payment with estimated incremental cost effectiveness ratios to determine whether a given treatment is cost effective relative to the alternatives.

In addition, two cost effectiveness acceptability curves were plotted to compare the following three different alternatives or scenarios with certain variables manipulated: (A) best care, followed by exercise plus fitness and health assessment at the Physical Activity Unit; (B) best care followed by the worst case scenario (in terms of salary, rate of participation, and efficacy); (C) the walking programme.

Four sensitivity analyses were performed to explore the robustness of estimations and how dependent the results were on estimates of unit costs per participant and efficacy. The first analysis examined the influence of the rate of participation in the programme since this could influence the technician's "productivity" based on the number of participants per unit of time provided by the technician. The second analysis estimated the cost of adding a permanent timetable of consultation, assessment or recruitment provided by the technician. To randomise all participants, the present trial only recruited participants prior to the beginning of the exercise programme; however, the widespread implementation of the programme would probably require permanent consultation. We estimated that exercise advisor schedule should increase 5 hours per week to attend the participants and new candidates to join the programme.

A third analysis explored the variations due to the salary changes of the technician since such changes are a major source of variability in economic studies [32].

Finally, the robustness of cost effectiveness was examined by exploring scenarios combining the influence of the variations in salary, rate of participation, and effectiveness from the lowest to the highest limit of the 95% CI.

## Results

### Response

Seventy-nine percent (127/160) of patients identified by general practitioners were recruited, but 20 of referred patients did not follow this referral; they were lost to follow-up or did not come to the research laboratory (Fig. 1). Eighty-six percent (55 of 64) of the participants in the walking group completed the programme. Fifteen to 22 patients attended each exercise group. At baseline, the intervention group was slightly less depressed, less overweight and younger than the control group, but these differences were not statistically significant (p > .05) (Table 1). The participants who were lost to follow-up (mainly because they had to care for a relative) were similar to those who completed the trial but a slightly higher percentage of them were moderately depressed. The participants in the control group who dropped out were similar to those who followed the trial but they were mainly living in an urban area. The social network in the rural area was stronger. Anxiety and depression, as measured by the EQ-5D, STAI and Geriatric Depression Scale, improved in the intervention group and mean BMI decreased (BMI mean change 1.2%; p = .003); these measures in the control group remained unaltered for the most part (Table 2).

**Table 1:** Baseline characteristics of patients allocated exercise programme or best care.

Characteristics	Exercise Completed Follow up	Best Care Completed Follow up	Exercise Lost to Follow up	Best Care Lost to Follow up
N	55	51	9	12
Age (years)	71(5)	74 (6)		
Living in rural areas (%)	67	65	67	33
Living alone (%)	24	18	22	17
Education, primary school or higher (%)	40	37	44	33
Income (€/month), (%):				
less than 360	4	3	11	0
360 to 600	89	91	89	92
more than 600	7	6	0	8
Daily smoking (1 or more cigarette/day)	1	0	0	0
Daily alcohol consumption (%)	11	4		
Physical activity standardised (%)	0	0	0	0
Overweight (%)	80	86	78	83
BMI	29.7 (4.2)	30.6 (4.3)		
Diabetes mellitus type II (%)	40	39	33	25
Moderate depressed (%)	33	39	44	33

**Table 2:** Health outcomes of the exercise programme compared to usual care

*Health outcome*	*Group*	*Baseline*	*Six months*	p*
Body Mass Index (kg·m^-2^)	exercise	29.7 (4.2)	29.4 (4.2)	.003
	Control	30.6 (4.3)	30.8 (4.3)	
Depression by Geriatric Depression Scale	Exercise	2.3 (2.5)	1.8 (2.3)	.001
	Control	2.6 (2.5)	2.9 (2.5)	
Anxiety by State Trait Anxiety Inventory	exercise	19.2 (11.2)	14.1 (9.0)	<.001
	Control	21.2 (10.4)	22.2 (9.8)	
Anxiety/Depression by EQ-5D	Exercise	1.4 (0.6)	1.2 (0.4)	.009
	Control	1.4 (0.6)	1.5 (0.7)	

*p of F by Analysis of Variance.

### Costs

The incremental cost of adding the exercise programme (Table 3), €2250, was related to the salary of the technician (10 hours per week; 25% of the total salary) over a 6-month period. Therefore, the mean incremental cost per participant, integrated in a group smaller than 30 participants, who attended three of the five available sessions per week, was €41.

**Table 3:** Incremental cost of the exercise programme compared to usual care.

***Concept***	unit*	***over 6 months (€)***	***Total (€)***
**Health system costs**			
Personnel†			
sport technician (25 weeks)	9 €/hour	2250	
Facilities (renting)	0 €/hour	0	
**Medication (no mean change were observed)**	Drug price	0	
**Consultation (no mean change were observed)**	Official price	0	
**Total health system perspective**			2250

. *Public cost in Euro in 2005.

† There is no marginal cost of adding or dropping-out participants in groups up to 30 persons.

### Health outcome and cost utility analysis

**Figure 2:**
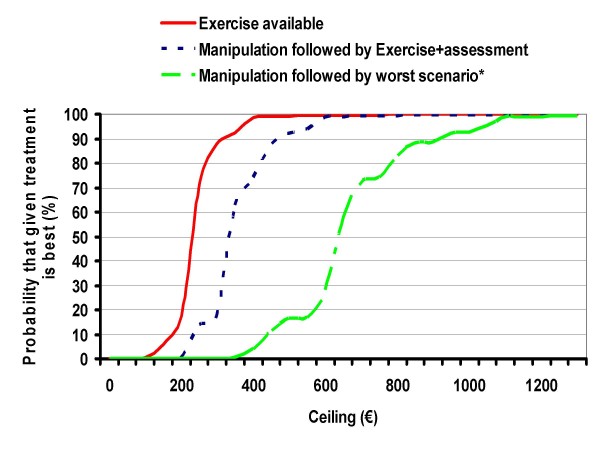
**Cost effectiveness acceptability curves**. * Worst scenario described as 30% higher salary, 30% lower participation rating and the effectiveness of lower limit of 95% confidence interval. – The efficiency threshold was set at 34729 €/QALY.

The interventional group improved more QALYs than the control group (Table 4) being adequate to perform a cost utility analysis. Each additional QALY gained by the exercise group cost less than €400. The cost effectiveness acceptability curves (Fig. 2) showed a 99.9% probability that the addition of the walking programme is an acceptable strategy if the ceiling of inversion is €600/QALY.

**Table 4:** The EQ-5D utilities of the exercise programme compared to usual care

*Alternatives*	*Best care in general * *Practice (n = 51)*	*Best care plus * *Exercise (n = 55)*
EQ-5D utility at baseline	0.542 (0.334)	0.688 (0.304)
EQ-5D utility at 6 months*	0.510 (0.196)	0.890 (0.178)
QALY over 6 months*	0.263 (0.132)	0.395 (0.121)
QALY difference versus best care†		0.132 (0.104 to 0.286)
Incremental cost per person (€)		41
Cost-utility (€/QALY) †		311 (143 to 394)

Expressed as mean (SD)

QALY = quality adjusted life year

*Mean (SD) estimated by analysis of covariance with adjustment for baseline EQ-5D score and then rounded to three significant figures.

† Mean (95% confidence interval estimated by bootstrapping).

### Sensitivity analysis

The sensitivity analyses are presented in Table 5. The first analysis manipulated the number of participants and their distribution in groups attended by the technician. This analysis showed a variation of cost-utility ratio from €311 to €462/QALY. In the second analysis, the predicted cost-utility of adding 5 hours per week, to assess and recruit new participants, was €462/QALY. The third sensitivity analysis focused on salary since this is a major source of cost variation related to location. If the salary was doubled the cost utility ratio was €621/QALY. The fourth analysis presented the best scenario or combination of the previously cited variables and the worst scenario. The estimated cost utility ratio ranged between €94/QALY of the best scenario and €871/QALY of the worst scenario.

**Table 5:** Sensitivity analyses by treatment group

Manipulation of variables	Incremental cost versus best care per person (€)	Cost utility ratio (€/QALY)
Number of participants:		
- 30% lower	58	439
- 30% higher*	47	356
- 50% lower or higher	41	311
- Distributed in 3 groups*	61	462
Including 5 h/week for assessment	61	462
Salary of technician:		
- 30% lower	29	220
- 30% higher	54	409
Best scenario of salary, participation and effectiveness†	27	94
Worst scenario of salary, participation and effectiveness‡	115	871

* Requires 5 additional hours of the technician

†Salary 30% lower + Participation 30 persons per group + QALY differential at higher limit of 95% confidence interval + distributed in two groups

‡Salary 30% higher + Participation 30% lower + QALY differential at lower limit of 95% confidence interval + distributed in three groups.

## Discussion

### Principal findings

The present study demonstrated that practitioners' exercise advice and the walking-programme efficiently enhanced the health-related quality of life in a high risk population, overweight or moderately depressed elderly women. This efficiency was based upon low-cost; however, a high recruitment rate was obtained by combining the medical advice of practitioners with the supervision of an exercise monitor.

### Strengths and weakness

To our knowledge, the current study is the first cost utility analysis of a walking exercise intervention with elderly females in primary care. It differed from traditional exercise referral schemes in that the physical activity advisors were specifically trained and employed for this programme [18]. In addition, walking, as well as strengthening and stretching exercises were used to enhance the feasibility of the programme in different environments. The most comparable study, by Munro et al. [33], analysed the cost utility of a strategy based on a letter from the research team inviting physically untrained persons older than 65 years of age to attend locally organised, free, twice weekly, exercise classes for 2 years. That study was conducted in Great Britain and the authors reported an incremental cost of €17174/QALY gained, using the utility index from the SF-36 and a mean cost per attendee per session of €9.1. In the present study we calculated an incremental cost of €311/QALY and €0.5/attendee per session. The differences between the study by Munro et al. and our study may be partly explained by: (a) the lower initial QALY in our population; (b) the gender of participants, who were predominantly female in the Munro study but exclusively female in our study; (c) the lengths of the programmes (2 years in the Munro study and 6 months in our study); and (d) the greater expense of the recruitment strategy of Munro et al. However, the impact of differences in programme length may (c, above) may have been minimized because most of the QALY increments in the Munro study were achieved in the first part of the 2-year period.

Previous studies have shown that recruiting elderly people in the general population to participate in exercise proves difficult, even when using different strategies [34,35]. In the framework of practice-based recruitment, Margitic et al. [19] reported that patient self-administered, office-based questionnaires were cheaper ($14/randomized participant) than patient mailings ($58) and direct telephone contact. Direct medical invitations to an exercise unit or office are easier to administer and less expensive; however, the rate of recruitment varies. Tully et al. [36] reported poor recruitment following contact by the general practitioner who invited patients to participate in an unsupervised home-based walking programme. Stevens et al. [20] achieved higher recruitment rates by inviting patients (45–74 years of age) to a consultation with an exercise development officer and offering a personalised combination of leisure-centre and home-based activities. However, our sensitivity analysis suggests that unit costs could be halved with a more effective recruitment strategy. Bell-Syer and Moffet [37] reported a 73% response rate to general practitioner reiterative invitations (an average of seven invitations) to participate in an exercise program for primary care patients with low-back pain. A recent review of exercise referrals in the United Kingdom [17] reported similar rates of recruitment by mail (50–60%) and low rates of maintenance without the continuous support of a technician. The strategy in the current study resulted in a recruitment rate of 79%; this strategy consisted of a specific targeted invitation from general practitioners to elderly women who were overweight or moderately depressed, offering a consultation with an exercise monitor and a supervised exercise programme. This high recruitment rate could be partially explained by the specificity of the invitation to older, overweight patients, who are usually more willing to participate than the general population [38], the active-management of general practitioners [39], and the offer of supervision in the exercise programme. Richert et al. [40] also demonstrated benefits of partnership and natural peer support in low-cost recruiting for enhancing physical activity. While recruitment rates were high in our programme, the retention rate of 86% was similar to previous supervised, group-based community strategies to promote exercise in the elderly population (80–90%) [6].

The NICE of the United Kingdom has recommended telephone support as an inexpensive alternative for maintaining the physically active lifestyle acquired by exercise referrals. However, more research is required to assess the comparative sustainability, retention, effectiveness and cost-effectiveness of exercise programmes (walking, cycling, advising or pedometer) that are longer than 10 weeks [14]. Benett et al. [41] has recently reported that the combination of a pedometer-based exercise program with monthly telephone support did not increase the level of physical activity in physically inactive persons. However, evidence suggests that the pedometer-based programme works well with more physically active persons [14,42].

### Unanswered questions

Exercise programmes targeted towards patients with particular diseases result in a demonstrable reduction in the use of health care services (frequentation, medication, etc.) [17,43,44]; however, exercise programmes targeted towards the general population have not been found to make any difference in the use of health care services in primary care [33]. The current study also failed to find changes in the use of primary health care services. The sample size was adequate to test the primary outcome, the utility of EQ-5D, but the sample size may have been insufficient to detect significant changes in the use of health services and medication. This relative reduction in the sample size was partially due to the selection criteria, which excluded patients with co-morbidities to allow us to focus the study on women with obesity and depression. The effects of the current programme on health system resources in a more wide-spread service could vary. However, the lack of change in frequency of consultations (consultation/month) in the short-term may be partially explained by the limits of supply and the management of "free" appointments in the general practices of the National Health System. Therefore, one should be cautious in generalizing these results to private care or more wide-spread services. In addition, this study may have been limited by a selection bias that favoured patients with low educational levels and low income, and who lived in medium sized cities and rural areas. As a result, more research is required to assess the efficiency of the current strategy in a more general population, with a longer programme, in different age or socioeconomic groups. The largest effect using the EQ-5D was detected in the dimension of anxiety/depression, so one could expect better cost-utility ratios in groups that include higher percentages of participants with problems in anxiety/depression.

## Conclusion

The current study presented a pragmatic and cost-effective strategy to enhance the level of physical activity in overweight or moderately depressed elderly women. The programme could be a cost-effective resource for helping patients increase physical activity, as recommended by general practitioners. Moreover, the present study could help decision makers enhance the preventive role of primary care and optimize health resources.

## List of abbreviations

BMI, body mass index; NICE, National Institute for Health and Clinical Excellence; QALY, quality-adjusted life years

## Competing interests

The authors declare that they have no competing interests.

## Authors' contributions

NG was involved in the conception, planning and design of this study, as well as the acquisition of data (but not the fitness testing), analysis and interpretation of data, and writing the manuscript. MCR was involved in organising this research, the acquisition of data and analysis and interpretation of data. JLG and JMG were involved in planning and organising the research and interpretation of data. EH assisted in the writing of the manuscript. All the authors read and approved the final manuscript.

## Pre-publication history

The pre-publication history for this paper can be accessed here:


